# The role of metabotropic glutamate receptor 5 in the pathogenesis of mood disorders and addiction: combining preclinical evidence with human Positron Emission Tomography (PET) studies

**DOI:** 10.3389/fnins.2015.00086

**Published:** 2015-03-18

**Authors:** Sylvia Terbeck, Funda Akkus, Laurence P. Chesterman, Gregor Hasler

**Affiliations:** ^1^School of Psychology, Faculty of Health and Human Sciences, University of PlymouthPlymouth, UK; ^2^Division of Molecular Psychiatry, Translational Research Center, Psychiatric University Hospital, University of BernBern, Switzerland; ^3^The Ansel ClinicNottingham, UK

**Keywords:** mGluR5, PET, mood disorders, addiction, anxiety

## Abstract

In the present review, we deliver an overview of the involvement of metabotropic glutamate receptor 5 (mGluR5) activity and density in pathological anxiety, mood disorders and addiction. Specifically, we will describe mGluR5 studies in humans that employed Positron Emission Tomography (PET) and combined the findings with preclinical animal research. This combined view of different methodological approaches—from basic neurobiological approaches to human studies—might give a more comprehensive and clinically relevant view of mGluR5 function in mental health than the view on preclinical data alone. We will also review the current research data on mGluR5 along the Research Domain Criteria (RDoC). Firstly, we found evidence of abnormal glutamate activity related to the positive and negative valence systems, which would suggest that antagonistic mGluR5 intervention has prominent anti-addictive, anti-depressive and anxiolytic effects. Secondly, there is evidence that mGluR5 plays an important role in systems for social functioning and the response to social stress. Finally, mGluR5's important role in sleep homeostasis suggests that this glutamate receptor may play an important role in RDoC's arousal and modulatory systems domain. Glutamate was previously mostly investigated in non-human studies, however initial human clinical PET research now also supports the hypothesis that, by mediating brain excitability, neuroplasticity and social cognition, abnormal metabotropic glutamate activity might predispose individuals to a broad range of psychiatric problems.

## Introduction

Glutamate is the primary excitatory neurotransmitter in the brain, and numerous researchers have suggested that it plays a significant role in various mental health and medical conditions. Indeed, researchers often mention the potential of developing ionotropic or metabotropic glutamate-based pharmacological treatment for numerous psychiatric disorders, in the form of either agonists or antagonists. Knowledge about the glutamatergic system has greatly advanced in the last decade, which has resulted from technological advances in receptor and transmitter imaging in humans. Even though there are a large number of experimental studies on glutamate intervention in various psychiatric disorders, there is a lack of systematic reviews that focus on combining the results of preclinical animal research studies with other neuroscience methods, such as human Positron Emission Tomography (PET). Thus, compared to previous reviews (Swanson et al., 2005; Pittenger et al., 2006; Kalivas, 2009; Brennan et al., 2012; Luykx et al., 2012; Riaza Bermudo-Soriano et al., 2012), this review examines human studies, mostly PET research, and combines those results with preclinical findings.

## Positron emission tomography (PET)

PET is a nuclear, sensitive, and non-invasive medical imaging technique used to image receptor distribution, concentration, and function. To identify human brain receptors, radio labeled receptor ligands (tracers) need to be developed. The scanner device detects gamma rays emitted by the tracer, which is introduced into the body. An mGluR5 PET tracer suitable for human studies was successfully developed at the Paul Scherrer Institute (PSI) in Villigen, Switzerland, and the Swiss Federal Institute of Technology (ETH) in Zurich, Switzerland. ABP688 is a non-competitive and highly selective antagonist, which binds to an allosteric site of the mGluR5. 11C-ABP688 showed high selectivity for mGluR5 and high uptake in receptor-rich brain regions. The first description of these characteristics of 11C-ABP688 in animals is previously published (Ametamey et al., 2006). We have seen promising results from studies performed in rats that use a beta-probe to estimate the kinetics of this tracer. Furthermore, when we performed the first PET study in humans to estimate the kinetics in humans (Ametamey et al., 2006) we found results comparable to those found in rat studies (Soares and Law, 2009). We have now successfully used the developed tracer for research on mGluR5 in healthy volunteers and psychiatric patients. PET techniques deliver information about the relative density of the receptors within the brain area examined. There is no information regarding the concentration of the neurotransmitter (i.e., whether high receptor density might lead to or be the consequence of increased or reduced neurotransmitter action). PET research delivers information regarding receptor level abnormalities in living human patients and thus helps to evaluate which receptors should be targeted with pharmaceutical treatment. On the contrary, Magnetic Resonance Spectroscopy (MRS) provides *in vivo* biochemical information about the tissue examined (Hasler et al., 2009) and thus provides information regarding the relative amount of glutamate metabolite in groups of patients. Findings from PET and MRS research can deliver additional evidence to pre-clinical animal studies. Animal research is the state of the art preclinical research method. Various animal models of psychiatric conditions have been established. Animal studies allow testing mechanical models, and testing of new pharmaceuticals in these models to reduce potential harm to humans. One disadvantage for animal research in psychiatry might, however, lie in the development of suitable animal models for different psychiatric conditions, specifically, conditions that involve internal or unique human cognitive aspects might lead to problems. Thus, a new pharmaceutical that shows improvement in animal behavior might not help human internal psychological aspects of the psychiatric condition.

This paper will thus examine if the findings from preclinical research in animals and human psychiatric PET research form a coherent view of mGluR5 involvement in mental disorders. This review will discuss only mGluR5 activity because extensive PET human data are available for this receptor type. mGluR5 delivers a promising target for drug development because a PET tracer can measure mGluR5 binding in humans, thus providing further insight into its functions in humans. Indeed, research suggested that drugs targeting the metabotropic glutamate group I receptors are among the most promising agents currently under development for the treatment of psychiatric conditions (Krystal et al., 2010).

In this review, we start with a brief introduction of the glutamate system and mGluR5, and then proceed to evaluate mGluR5 involvement in anxiety and mood disorders, and addiction by comparing previous preclinical trials with recent PET studies. We will then describe an approach how mGluR5 based interventions might be efficient by contributing to changes in learning and social functioning, and by decreasing excitability within various brain regions.

## The glutamatergic system and mGluR5

**Figure 1:**
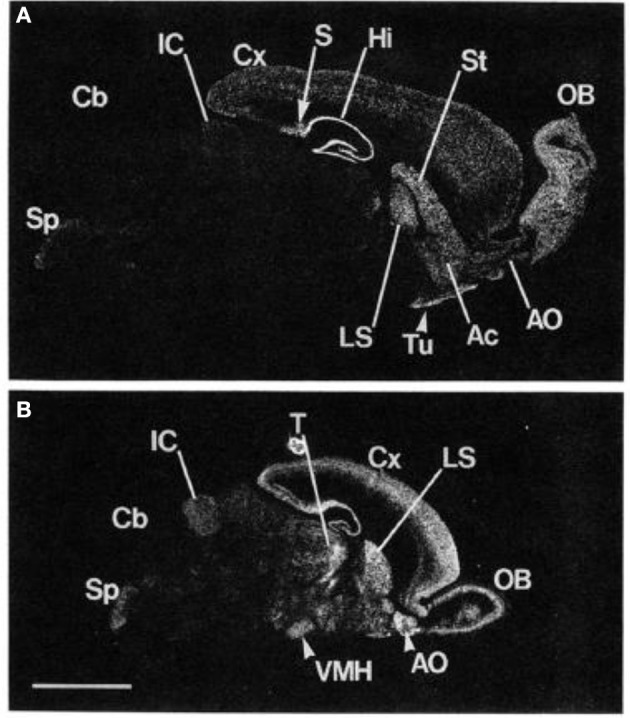
**Localization of mGluR5 mRNA in the adult and the 6-day-old rat brains by *in situ* hybridization**. Negative film images of sagittal section of the adult rat brain **(A)** and the 6-day-old rat brain **(B)** are shown. *OB*, main olfactory nucleus; *Ac*, accumbens nucleus; *Tu*, olfactory tubercle; *St*, striatum; *Hi*, hippocampus; *S*, subiculum; *Cx*, cerebral cortex; *LS*, lateral septal nucleus; *IC*, inferior colliculus; *Cb*, cerebellar cortex; *Sp*, spinal trigeminal nucleus; *T*, thalamus; *VMH*, ventromedial hypothalamic nucleus. *Scale bar*, 4 mm. From Abe et al. (1992). Note: the figure and its legend is reproduced with permission.

Glutamate regulates central nervous system function through the actions of ionotropic and metabotropic receptors. The involvement of glutamate in various psychiatric and medical conditions has been intensively examined. However, earlier work mostly focused on ionotropic glutamate receptors. In contrast to the fast and direct actions of ionotropic receptors, the three groups of metabotropic (mGlu) receptors modify neuronal activity through G-protein coupled signaling. Groups of mGluRs are distinguished by their pharmacological and intracellular signaling properties. mGluR5, which was first cloned in animals in 1992 and a few years later in humans, belongs to group I metabotropic receptors (Olive, 2005). Its actions are predominantly excitatory (Meldrum, 2000). Cleva and Olive (2011) described strong links and receptor interactions between mGluR5 and NMDA receptor, suggesting that mGluR5 might also be extensively implicated in mediating neural plasticity as well as learning and memory processes. In addition, there is some evidence that mGluR5 activation enhances GABA, especially in the nucleus accumbens (Hoffpauir and Gleason, 2002). Thus, it is suggested that metabotropic glutamate receptor activity can modulate excitatory and inhibitory (GABA) signaling pathways. High mGluR5 receptor density was identified primarily in the forebrain regions, striatum, and limbic regions including the amygdala and hippocampus (Swanson et al., 2005). Using advanced molecular biological techniques to determine mGluR5 mRNA expression in the rodent brain, research determined that regions of the olfactory bulb, dorsal striatum, nucleus accumbens, lateral septum, and hippocampus show the highest levels of mGluR5 expression (Abe et al., 1992) (See Figure 1).

Pre-synaptic mGluR5 receptors were found to participate in the regulation of synaptic plasticity and changes in neuronal excitability to maintain homeostasis (Schoepp, 2001). Importantly, significant mGluR5 expression can already be determined at 9 Gestational Week (GW) prenatal (Boer et al., 2010). Additionally, mGluR5 expression was found to be much higher in younger animals than in adults (Romano et al., 1996), suggesting that early intervention targeting the mGluR5 might have preventive effects in neurodevelopmental disorders.

Because of its functions in different neuronal processes, gross abnormalities in the glutamate system lead to severe neurological impairment (e.g., seizure disorder), whereas smaller changes can contribute to various psychiatric conditions (Yüksel and Öngür, 2010). Efforts to develop drugs that selectively target mGluR5 began in the late 90s. To date, various mGluR5 agonists and antagonists have been developed (See Lea and Faden, 2006 for further information regarding specific drug development). For example, 3-[2-methyl-1,3-thiazol-4yl]ethynyl]pyridine (MTEP) is a highly selective and potent non-competitive mGluR5 receptor antagonist that achieves full receptor occupancy 1 h after injection in rats with a dose of 10 mg/kg (Anderson et al., 2003). Table 1 gives an overview of some mGluR5 drugs used in animals and those previously used in the initial clinical human trials.

**Table 1:** **Selection of mGluR5 acting pharmaceuticals**.

**mGluR5 Drug**	**Mechanism**	**Usage**	**References**
MTEP	mGluR5 antagonist	Animal research	e.g., Gass et al., 2009; Koros et al., 2007
Acamprosate	mGluR5 antagonist	Human trials	e.g., Erickson et al., 2011
Memantine	mGluR5 antagonist	Human trials	e.g., Erickson et al., 2009
AFQ056	mGluR5 antagonist	Human trials	e.g., Berg et al., 2011
Fenobam	mGluR5 antagonist	Human trials	e.g., Berry-Kravis et al., 2009

## Psychiatric disorders and glutamate based intervention in humans

Psychiatric disorders are very heterogeneous and co-morbidity is common. However, common psychiatric pharmacological treatments are based on relatively few pathophysiological mechanisms, for example, increasing monoamine availability in anxiety and depression. Thus, there is an urgent need to improve and advance psychiatric treatments, and metabotropic glutamate based pharmacological intervention is a promising development in this regard (Agid et al., 2007).

Most human trials have been conducted on cases of Fragile X Syndrome (FXS). The mGluR5 theory of FXS posits that the lack of fragile X mental retardation protein (FMRP) results in excessive glutamatergic signaling via mGluR5 (Bear et al., 2004). This leads to increased local mRNA translation at the synapse because FMRP is not present to regulate the process. Finally, this weakens the synapse and results in an increased number of longer immature dendritic spines, which could explain the intellectual disability found in FXS patients. This disability is associated with mood and anxiety symptoms and typically present with features that are common in autism spectrum disorder, including delays in speech and language development, impaired theory of mind, and impaired social and emotional processing, as well as repetitive behavior (Garber et al., 2008). Preliminary and indirect evidence that mGluR5 antagonist can improve sociability in FXS (Burket et al., 2014) raises hopes that drugs targeting the mGluR5 might be of clinical use in prevalent psychiatric conditions associated with impaired systems for social processes such as autism, schizophrenia, and depression. Furthermore, the observable repetitive behavior phenotype in FXS might suggest a shared pathophysiology among other psychiatric disorders such as obsessive-compulsive disorder (OCD) and addiction.

Although there has been an increase in the scientific knowledge and research on mGluR5, drug development efforts have been relatively unsuccessful (Agid et al., 2007). Drugs that target the ionotropic receptors usually produce numerous side effects and current drug development strategies have not yet produced selective targets for ionotropic receptors that might reduce potential side effects. For instance, ionotropic receptor antagonists produce side effects in humans that include memory impairment, psychotic episodes, and strokes (Swanson et al., 2005). The unfavorable side effects might occur because ionotropic glutamate receptors have a ubiquitous distribution, whereas metabotropic receptors are more uneven and selectively distributed (Krystal et al., 2010). As a result, recent drug development has focused on compounds targeting metabotropic receptors assuming that such drugs will be associated with fewer side effects than those that bind to the fast-acting ionotropic receptors.

In the following sections, we will review evidence from human PET studies regarding mGluR5 involvement in mood disorders and addiction and compare those findings to animal studies. Additionally, we will describe some brain locations for mGluR5 activity in humans and finally suggest an approach how direct and indirect mGluR5 activity might be involved in human psychiatric syndromes.

## mGluR5, pathological anxiety, and mood disorders

### Pathological anxiety

Pathological anxiety occurs in anxiety disorders, including generalized anxiety disorder, panic disorder (the most prevalent psychiatric conditions worldwide, First and Gibbon, 1997), but also in other prevalent psychiatric conditions such as depression and obsessive-compulsive disorder (OCD). These psychiatric conditions cause significant impairment in both social and occupational functioning, leading to health cost burdens and patient suffering (First and Gibbon, 1997). Generally, anxiety might be associated with excessive brain excitability (Harvey and Shahid, 2012).

#### Preclinical neurobiological research

Findings from a large number of preclinical animal trials have determined the effect of mGluR5 antagonist treatment in anxiety. Swanson et al. (2005) reviewed animal studies on drugs targeting the mGluR5 on anxiety-like behaviors. They concluded that mGluR5 antagonistic treatment mostly led to anxiolytic responses in experimental animals. In particular, effects such as reduced fear conditioned freezing, increased shock and punishment acceptance, and increased social interactions were observed. For example, a single dose of 2-methyl-6-(phenylethynyl)pyridine (MPEP) increased the amount of time that rats spent in the open arm of an experimental maze, without affecting planning or motor behavior (Tatarczyńska et al., 2001). Krystal et al. (2010) reviewed preclinical animal studies that examined mGluR5 antagonists (MTEP, MPEP, fenobam) in mouse models of anxiety. These studies used different outcome measures, such as extinction of fear conditioning and responses in the elevated plus maze, to assess the effectiveness of drug treatments. Of the studies examined, 88.45% reported an anxiolytic effect with mGluR5 antagonists (Krystal et al., 2010). More recently, another review on anxiety research in animal models that examined the effect of ionotropic and metabotropic glutamate receptor antagonist intervention was published (Riaza Bermudo-Soriano et al., 2012). Regarding mGluR5, the authors listed 43 animal studies of anxiety, and all but two demonstrated anxiolytic effects.

#### Human studies

Initial evidence for the hypothesis that glutamate function is abnormal in anxiety disorders came from MRS research. For instance, using single-voxel high-field 1H-magnetic resonance spectroscopy, the researchers found that compared to healthy controls, patients with social anxiety disorder showed significantly higher glutamate levels in the anterior cingulate cortex (ACC) (Phan et al., 2005). Additionally, research found increased global glutamate concentration in 10 patients with social phobia (Pollack et al., 2008). As stated before, these studies, however, could not determine which glutamate receptors were associated with excessive glutamate activity.

Employing PET research methodology, we recently were the first to show relationships between mGluR5 and anxiety, in Major Depressive Disorder (MDD), and OCD. In one study, we researched mGluR5 Distribution Volume Ratio (DVR) in 10 patients with OCD and 10 healthy controls using [11C]ABP688 PET (Akkus et al., 2014). We employed the Yale-Brown Obsessive-Compulsive Scale (Y-BOCS) as a clinical measure of OCD symptom severity. We observed significant positive correlations between Y-BOCS obsession scores and mGluR5 DVR in regions of the amygdala, ACC, and medial orbitofrontal cortex (Akkus et al., 2014). These brain areas have previously been implicated in OCD pathophysiology. Indeed, structural brain abnormalities in the amygdala, the ACC, and the orbitofrontal cortex consistently have been associated with OCD (Rosenberg and Keshavan, 1998; Szeszko et al., 2008; Van den Heuvel et al., 2009). Given that structural imaging studies have provided evidence for a positive correlation between OCD severity and gray matter volume (Zarei et al., 2011), increased mGluR5 binding in OCD may reflect an increased density of neurons. Although in DSM-5 OCD is not considered anymore as an anxiety disorder, most OCD patients experience anxiety symptoms. In a relatively large clinical sample, we have previously shown that OCD patients with obsessions have a particularly high prevalence of anxiety symptoms and disorders (Hasler et al., 2005). In this paper, we suggested that obsessions that involve stress, anxiety, or conflict thus might be associated with increased glutamatergic neurotransmission in the amygdala, ACC, and orbitofrontal cortex.

Taken together, the findings from recent studies using different research methodologies support the hypothesis that glutamate function is abnormal in key areas of the limbic system in mood disorders related to anxiety. The abnormal function is likely to also be related to the mGluR5 receptor. Since, pre-clinical, as well as PET research showed a consistent pattern of results, we suggest that antagonistic mGluR5 treatment would produce significant anxiolytic effects in patients suffering from pathological anxiety.

### Major depressive disorder (MDD)

#### Preclinical neurobiological research

In their review, Krystal et al. (2010) described the outcomes of eight studies investigating the mGluR5 antagonists MTEP and MPEP in animal models of depression. Compared to the treatment success rates for anxiety disorders, the authors reported that only 62.5–75% found a clear antidepressant effect, even though treatment with the ionotropic NMDA antagonist ketamine has been shown to lead to rapid antidepressant effects, even in treatment resistant patients (Pittenger et al., 2006).

#### Human studies

A recent review reviewed 13 MRS studies of mood disorders. The authors reported that these studies consistently found evidence that glutamate was reduced in MDD (Hasler et al., 2007; Yüksel and Öngür, 2010). In particular, reduced glutamate levels in the ACC, left dorsolateral prefrontal cortex, dorsomedial prefrontal cortex, ventromedial prefrontal cortex, amygdala, and hippocampus were found. A later review on studies that used MRS to examine glutamate in MDD confirmed that glutamate concentration in the ACC was consistently reduced (Luykx et al., 2012). In the occipital cortex, increased glutamate were found by some researchers, which were highest in the melancholic subgroup of patients with MDD (Sanacora et al., 2008).

In a previous study, we obtained PET images of mGluR5 receptor binding in 11 unmedicated subjects with MDD and 11 healthy controls (Deschwanden et al., 2011). We found decreased regional mGluR5 binding in the prefrontal cortex, cingulate cortex, insula, thalamus, and hippocampus of the patients suffering from depression. Additionally, the severity of depression negatively correlated with mGluR5 binding in the hippocampus. We suggested that these findings indicate reduced mGluR5 neurotransmission in depression, possibly as a result of basal or compensatory changes in the glutamate system activity. In addition, we examined the amount of mGluR5 expression in post-mortem brain samples of 15 depressed subjects and 15 matched controls (Deschwanden et al., 2011). We observed reduced mGluR5 expression in the prefrontal cortex in samples obtained from depressed individuals. Reduced NMDA receptor expression in the post-mortem brain of depressed patients also was reported (Feyissa et al., 2009). Indeed, it was previously suggested that the antidepressant properties of mGluR5 antagonists may involve inhibition of NMDA receptor. This might mediate neurotransmission and/or induction of brain-derived neurotropic factor gene expression in the hippocampus (Legutko et al., 2006). Further, a recent study showed that sleep deprivation increases mGluR5 availability in healthy humans (Hefti et al., 2013). In anterior cingulate cortex, insula, medial temporal lobe, parahippocampal gyrus, striatum, and amygdala, this increase correlated significantly with the efficacy of sleep deprivation as reflected by increased subjective sleepiness. This study suggests that an increase in mGluR5 may be a neurobiological mechanism explaining sleep deprivation's high antidepressant efficacy. Preclinical studies confirm our hypothesized association between mGluR5, sleep and depression. In particular, a study in mGluR5 knock-out mice provides important evidence that mGluR5 is involved in shaping the stability of NREM sleep-REM sleep state transitions, NREM slow wave activity and homeostatic response to sleep loss (Ahnaou et al., 2015).

To summarize, there is convergent evidence from animal, postmortem, MRS, and PET studies that the central glutamate system is importantly involved in the pathophysiology of MDD. However, the evidence suggests that mGluR5 antagonism might not directly help patients suffering from MDD. This is in accordance with our finding of reduced mGluR5 expression in MDD patients. It could be speculated that drugs that target the mGluR5 system may be particularly useful in depressed patients with comorbid anxiety, addiction disorders and/or impaired circadian rhythms. Further, impairments in the systems for social processes are commonly associated with MDD. Poor social skills have appeared to be an important risk factor for depression (Segrin, 2000). Such deficits including paralinguistic and linguistic behaviors, and impairments in facial expression, gaze, posture and gesture, that are comparable to those observed in FXS and autism spectrum disorder. Furthermore, regarding social skills, experiments in mice exposed to chronic social stress showed that Homer1/mGluR5 coupling was disrupted, suggesting that mGluR5 night moderate the depressive vulnerability to social stress (Wagner et al., in press). Additionally, in mouse models of social deficits, mGluR5 suppression has led to normalization of social interactions (Chung et al., 2015). Together, these studies provide preclinical evidence that mGluR5 plays important roles in the social causes of depression and the social deficits frequently observed in depressed patients. As a result, drugs targeting the mGluR5 may play an important role to prevent the development of depression in youth with social deficits and may help to treat social deficits and low psychosocial functioning in patients with MDD.

## mGluR5 and addiction

Addiction is characterized by continued drug intake despite negative consequences, repeated unsuccessful attempts to stop or reduce drug use, and symptoms of tolerance and withdrawal. Although the dopamine system plays a key role in acute reward processing (Kalivas and Volkow, 2005), there is growing evidence implicating glutamatergic neurotransmission in addiction (Krystal et al., 2010).

### Preclinical neurobiological research

In 2001, a seminal study on mGluR5 and addiction was published (Chiamulera et al., 2001). The authors showed that mice lacking the mGluR5 receptor failed to acquire intravenous cocaine self-administration despite showing increased extracellular dopamine levels in the nucleus accumbens following acute injection. Numerous follow-up animal studies have demonstrated that the mGluR5 receptor antagonists MPEP and MTEP reduce self-administration of addictive drugs such as cocaine and nicotine (Kalivas, 2009). Olive (2005) reviewed animal studies related to drug addiction and suggested that there is evidence that mGluR5 might be involved in development, reward perception, and relapse of drug use such as that of cocaine, morphine, nicotine, and ethanol. The authors described evidence from animal studies that mGluR5 antagonists reduced self-administration of the drug, as well as subsequent drug-seeking behavior. For example, recently, it was discovered in a baboon model of binge-eating disorder that MTEP decreased candy consumption without altering candy-seeking behavior (Bisaga et al., 2008). Research suggested that the decrease of food intake associated with mGluR5 receptor antagonist use might be related to a reduction in the rewarding value of the reinforcing stimuli (Bisaga et al., 2008). Additionally, research reported significant increases in mGluR5 mRNA levels in the nucleus accumbens and dorsolateral striatum after repeated cocaine administration in experimental rats (Bisaga et al., 2008).

### Human studies

**Figure 2:**
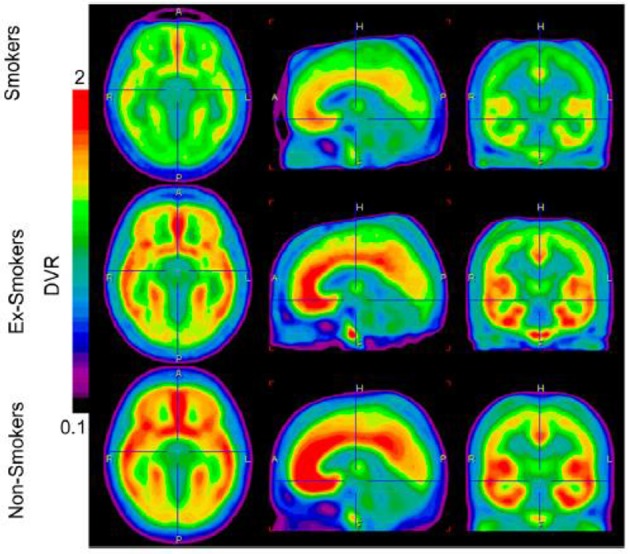
**Image display the average brain uptake of mGluR5 DVR in the three diagnostic groups**. The brain uptake is visible reduced in smoker and ex-smokers groups. Images are calculated by PMOD software version 3 (PMOD Technologies). From Akkus et al. (2013). Note: the figure and its legend is reproduced with permission.

Using autoradiography in post-mortem brain tissue samples obtained from patients suffering from alcohol disorders and healthy controls, research found 30–40% higher mGluR1/5 binding density in the hippocampus and striatum in patients suffering from alcohol disorders (Kupila et al., 2012). This finding suggested that mGluR5 receptor density might be increased in some brain areas of alcohol-addicted patients. Recently, we used PET to measure mGluR5 receptor binding in the brains of individuals with nicotine addictions (Akkus et al., 2013). We found a global reduction in mGluR5 DVR in the gray matter of 14 smokers compared to non-smokers (See Figure 2). The most prominent reductions were found in the right and left medial orbitofrontal cortex. We suggested this decrease in mGluR5 receptor binding might be a long-lasting adaptation to chronic increases in glutamate induced by chronic nicotine administration. This adaptation seems to be specific for nicotine dependence since it has not been found in chronic non-smoking cocaine users (Hulka et al., 2014).

**Figure 3:**
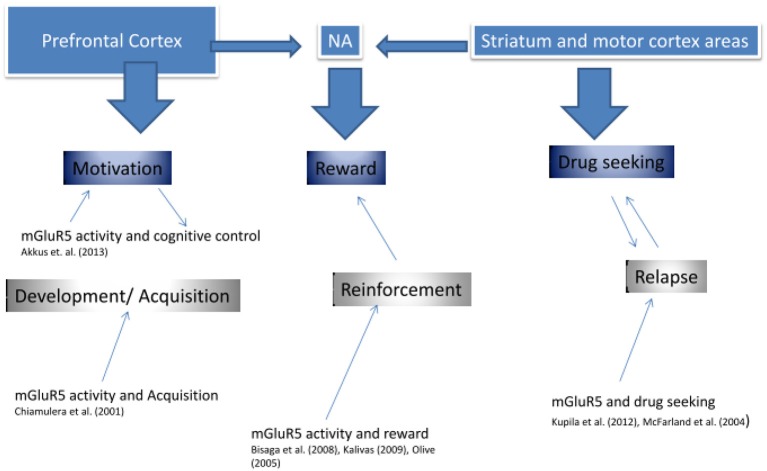
**mGluR5 involvement in addiction**.

Indeed, it has been suggested that mGluR5 downregulation represents a compensatory neuroadaptation (Kalivas, 2009), an enhancer of drug-induced reward (Rutten et al., 2011), or a factor mediating the effects of contextual cues in conditioned behavioral responses (Tronci et al., 2010). Figure 3 presents an approach of mGluR5 dysfunction in addiction.

As seen in Figure 3, previous research supports the idea that mGluR5 is involved in three key stages of addiction, in the development and acquisition, in the reinforcement value of the drug, as well as in addiction relapse. It might be suggested that each function is predominantly described by different brain regions, which also show high mGluR5 receptor density, and which have been shown to display reduced mGluR5 density in our PET research (Deschwanden et al., 2011). Kalivas (2009) developed an extensive glutamate model of addiction. The author suggested that addiction was associated with dysfunction in glutamate homeostasis between key areas of the corticostriatal brain circuitry, including the amygdala, nucleus accumbens (NA), prefrontal cortex, and motor cortex. As we suggest in Figure 3, firstly (“Motivation component”), the prefrontal cortex might mediate motivation and cognitive control during the initial phases of drug addiction development. The preclinical study by Chiamulera et al. (2001) suggests that mGluR5 is required in this phase of addiction development by mediating the rewarding properties of drugs of abuse. Secondly (“Reward component”), the Nucleus Accumbens (NA) has been shown to influence the rewarding value of the drug, not only via dopamine but also via mGluR5 activity (Bisaga et al., 2008). In particular, impairment in glutamate transmission between the prelimbic cortex and the NA was suggested, such that drug seeking was initiated by a greater reliance on learned behavior associated with the NMDA/mGluR5 receptor system (Kalivas, 2009). And thirdly (“Drug Seeking component”), some research supports that mGluR5 is involved in higher reliance on motor processes—reducing cognitive control—via striatum (Kupila et al., 2012). Our own published and unpublished research suggests that mGluR5 downregulation is associated with increased risk of relapse in ex-smokers (Akkus, PNAS). This downregulation may be a pathogenic or an insufficient compensatory change. Taken together, the current research suggests that drugs targeting the mGluR5 may improve treatments of addiction disorders in various stages of their development.

## Discussion

The reviewed evidence suggests high involvement of mGluR5 within anxiety disorders, OCD, MDD, as well as addiction, and that treatment with mGluR5 targeting pharmaceuticals might be beneficial also in humans. However, the pathogenesis of mood and anxiety symptoms associated with OCD may differ from mood and anxiety symptoms unrelated to OCD. As a result, the presence of obsessive-compulsive symptoms may be an important predictor of the antidepressant and anxiolytic response to drugs targeting the mGluR5. Furthermore, we have suggested that drugs targeting the mGluR5 system may help to increase resistance to social stress and improve social deficits in depression. Since social stress is by far the most important non-genetic risk factor of depression and social deficits are closely linked to reductions in social functioning and quality of life, these findings from animal studies are of high scientific and clinical relevance.

Recently, the National Institute of Mental Health (NIMH) has initiated a novel, state-of-the art project: The Research Domain Criteria (RDoC). This reflects the implementation of NIMH Strategy 1.4 “Develop, for research purposes, new ways of classifying mental disorders based on dimensions of observable behavior and neurobiological measure.” (http://www.nimh.nih.gov/research-priorities/strategic-objectives/strategic-objective-1.shtml). This initiative was the results of restructuring rigid DSM categories, most of which were also developed before neuroscience research (Morris and Cuthbert, 2012). RDoC describes five domains or constructs; Negative valence system, Positive valence system, Cognitive Systems, Systems for social processes, Arousal/regulatory systems. In accordance with the reviewed evidence on the involvement of mGluR5 in MDD and OCD, we propose that mGluR5 activity might directly be associated with the negative valence system, which involves the observable factors of fear, threat, sustained threat, loss, and frustrated non-reward. Thus, as reviewed studies suggest, antagonistic treatment with mGluR5 should reduce those observable symptoms. Further, we suggest that mGluR5 treatment might also be beneficial in “positive valence disorders” such as addiction and depression, via abnormal mGluR5 activity on brain structure and function related to the glutamatergic NMDA receptor that is functionally linked with mGluR5 and importantly involved in reward learning. For example, Simonyi et al. (2010) reviewed numerous animal studies which employed mGluR5 receptor antagonists in knockout mice to determine the role of mGluR5 in learning and memory. Inhibitory learning, such as passive avoidance learning, is a well-established task in animal models that is used to study hippocampal learning processes, and has been shown in numerous studies to be dependent on the mGluR5 receptor (Simonyi et al., 2010). For instance, research demonstrated hyperexpression of mGluR5 protein in CA3 during short- and CA1 long-term potentiation in rats (Riedel et al., 2000). Hyman (2005) presented a biological model of addiction that incorporates abnormal neural processes of learning and memory forming the basic elements of addiction. The authors proposed that long-term potentiation, which includes, alterations in the availability of glutamate receptors, and regulation of gene expression as potentially important mechanisms for the drug-induced alterations found in the abnormal circuits associated with drug addiction. Finally, the studies on mGluR5 and sleep homeostasis (Hefti et al., 2013; Ahnaou et al., 2015) suggest an important role of mGluR5 in RDoC's arousal and modulatory systems domain.

**Figure 4:**
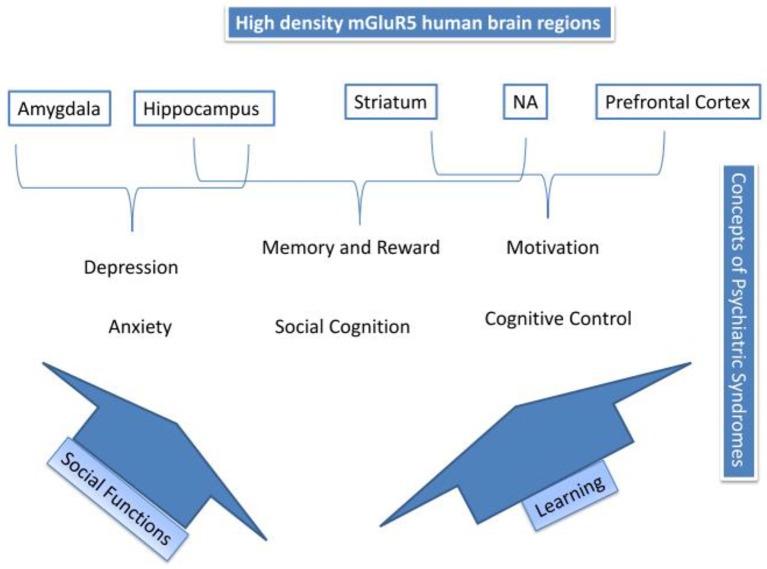
**Summary of mGluR5 mechanisms**.

Figure 4 summarizes the processes proposed to mediate the actions of mGluR5 in mood disorders and addiction. The top of Figure 4 depicts high-density mGluR5 brain regions; amygdala, hippocampus, striatum, NA, and prefrontal cortex. Psychiatric syndromes are matched to those regions. mGluR5 activity, suggested in the amygdala, might mediate primary emotional arousal, such as anxiety and depression. We have described how two possible pathways; social functioning, and learning, might mediate the other processes. Thus, mGluR5 activity has shown to be involved in learning, and thus, via activity in the hippocampus, NA, and striatum might be involved in memory and reward, cognitive control and motivation, as also implicated in addiction (See also Figure 3). Finally, we have suggested how mGluR5 may be related to the social stress response and social deficits, which might be relevant for a broad range of psychiatric conditions. Table 2 gives an overview of the RDoC area of impairment, the associated clinical picture, and mGluR5 involvement.

**Table 2:** **RDoC and mGluR5**.

**RDoC**	**Sample mechanism**	**Brain regions**	**Possible symptoms of mGlu5 dysfunction**	**Psychiatric disorders**
Positive valence system	mGluR5 mediates rewarding effects	Striatum, Orbitofrontal cortex	Drug addiction, adhedonia	Nicotine dependence, Substance misuse, depression
Negative valence system	mGluR5 regulates the response to stress /threat	Amygdala, ventromedial PFC	Anxiety, decreased stress tolerance	Depression, OCD, anxiety disorders
Systems for social processes	Weakening of synapse formation	globally	Social deficits	Fragile X syndrome, autism, depression
Arousal/regulatory system	mGluR5 mediates brain excitability	Limbic system	Emotional arousal, impaired circadian rhythms	Anxiety disorders, OCD, depression

## Concluding remarks

This review described the involvement of mGluR5 in mood disorders, OCD and addiction, and compared preclinical with human research, specifically PET research. We thus compared different methodological approaches, such as animal research, MRS, and PET studies. We suggest that a strong direct anxiolytic effect would be present if mGluR5 antagonistic treatment was initiated in human clinical trials (RDoC). mGluR5 overactivity has also been reported in FXS, which is characterized by important social deficits. As a result, mGluR5 activity may not just normalize activity in the negative valence systems and arousal systems, but also attenuate impairments in the systems for social processes (RDoC). This is of high clinical importance because poor social functioning is an important outcome of prevalent psychiatric conditions such as OCD, depression and addiction, leading to enormous personal suffering and important indirect costs for the society. And finally, mGluR5 was also shown to have a significant involvement in drug addiction, suggested to be mainly caused by increasing the reward value of the drug. mGluR5 antagonistic intervention would thus be most effective for the treatment of pathological anxiety and addiction, and to improve social stress resiliency and social functioning.

### Conflict of interest statement

Dr Terbeck, Dr Chesterman, and Dr Akkus have no conflict of interest. Dr Hasler has received grant funding from Novartis, which is producing and testing drugs targeting mGluR5.
